# Metabolic inflexibility of mitochondria: beneficial for the fitness of regenerating liver cells

**DOI:** 10.1038/s41392-024-01959-1

**Published:** 2024-09-09

**Authors:** Josef Ecker, Sarah Brunner, Klaus-Peter Janssen

**Affiliations:** 1 Institute of Clinical Chemistry and Laboratory Medicine, Functional Lipidomics and Metabolism Research, University Hospital Regensburg, Regensburg, Germany; 2 Department of Surgery, School of Medicine and Health, Technical University of Munich, 81675 Munich, Germany

**Keywords:** Gastrointestinal diseases, Transdifferentiation, Metabolic disorders

In a recent manuscript in *Science*, Wang and coworkers discovered that metabolic inflexibility is required for proper mitochondrial function during liver regeneration.^1^ This study deepens our understanding of liver metabolism, stem cell biology and adult tissue renewal, suggesting that metabolic flexibility, generally assumed to be a selective advantage, may actually be detrimental for mitochondrial function in the regenerating liver.

**Fig. 1:**
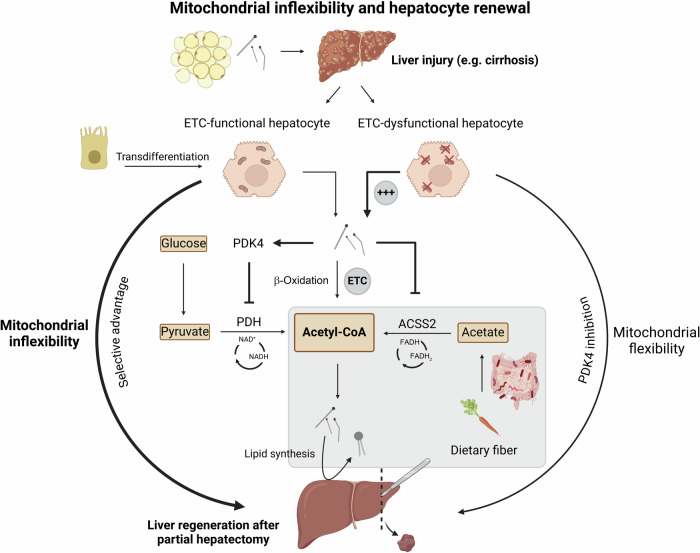
Mitochondrial inflexibility and hepatocyte renewal. During liver injury and regeneration, fatty acids released from adipose tissue fuel liver mitochondria to maintain acetyl-CoA levels. Cholangiocytes (upper left) have the capacity to undergo transdifferentiation into hepatocytes, and phospholipids are generated in the liver from acetate (Short chain fatty acids derived from microbiota-metabolized dietary fiber), both of which further promote liver regeneration. Mitochondria with ETC dysfunction upregulate PDK4 and inhibit ACSS2 expression, ultimately blocking acetyl-CoA generation and stopping proliferation of dysfunctional hepatocytes. Mitochondrial inflexibility therefore selects for ETC-functional healthy mitochondria and promotes liver recovery after partial hepatectomy. Conversely, PDK4 inhibition (mitochondrial flexibility) increases the proliferation of ETC-dysfunctional mitochondria. Created with BioRender

Whereas these findings appear surprising in the light of traditional metabolic concepts, they offer a detailed view into the regulatory networks that ensure that only healthy liver cells with functional mitochondria contribute to regeneration. The study has considerable relevance for human health, since primary or metastatic cancer often affects the liver. Moreover, chronic hepatitis, toxins, or metabolic stress can lead to the development of fibrosis, cirrhosis, and chronic conditions like alcoholic steatohepatitis (ASH) and metabolic dysfunction-associated steatohepatitis.^2^ Thus, liver diseases rank among the global health burdens, and research efforts are needed to better understand the processes that govern the self-renewal capacity of the adult liver (Fig. 1).

As a radical yet potentially curative treatment option, it would appear logical to replace non-functional livers by transplantation. However, the continuing shortage of donor organs for liver transplantation has often been acknowledged. In the clinical context, a major treatment option therefore is partial hepatectomy (PHx), based on surgical removal of cancerous tissue with the objective of sparing as much of functional liver parenchyma as possible to allow for its self-renewal. Up to 75% of healthy liver tissue can be safely removed, indicative of the stunning regenerative capacity of the liver in adults.^2^ The outcome of this procedure is confounded by diverse pathologies and by the type of intervention itself, but five-year survival rates of around 50% can be obtained.^2^

The processes underlying liver regeneration are not fully understood, and regenerative capacities are drastically limited under disease conditions, such as metabolic dysfunction-associated fatty liver disease (MAFLD). In order to follow these processes in depth, the authors have tackled mitochondria, the central regulators of bioenergetics and metabolic powerhouses of eukaryotic cells. They focused on the crucial components in mitochondria, the electron transport chain (ETC). This complex multi-protein machinery is composed of four inner mitochondrial membrane complexes and two electron carriers, coenzyme Q and cytochrome c, which oxidize reducing equivalents (e.g., NADH). The resulting electrochemical proton gradient enables ATP-synthase to generate energy within cells. Of note, ETC dysfunction has been involved previously in liver pathologies by the same working group.^3^

By default, the regenerating liver can be repopulated from preexisting hepatocytes that enter the cell cycle and proliferate. As a backup mechanism to compensate for a lack of proliferating hepatocytes, the epithelia lining intrahepatic biliary ducts (cholangiocytes or biliary epithelial cells) can transdifferentiate into hepatocytes via transitional liver progenitor states, and thus help to regenerate the liver parenchyma. However, how is it ensured that only metabolically healthy liver cells with fully functional mitochondria contribute to regeneration? Moreover, how are cells with defective ETC excluded from this process, to avoid clonal expansion of “unfit” hepatocytes?

The Mishra lab has revealed in an elegant and compelling set of experiments, based on a series of genetic mouse models, a selective mechanism that prevents hepatocytes containing defective ETC to propagate. This process selects for healthy mitochondria, which finally leads to successful and effective liver regeneration.

The beneficial mechanism uncovered in this study has been termed “metabolic inflexibility”. This may appear counterintuitive since the flexibility of the mitochondrial metabolism is essential for mammalian organisms to adapt to different food sources or to survive fasting periods. Moreover, obesity and type 2 diabetes are commonly associated with “metabolic inflexibility”. However, the “inflexibility” of mitochondria actually helps to maintain hepatocytes healthy and ultimately boosts liver regeneration. Under physiological conditions, acetyl-CoA can be generated either from glycolytic breakdown of carbohydrates, or via β-oxidation from fatty acids, and enters the citric cycle in healthy mitochondria to generate energy in the form of ATP and GTP.

Applying stable isotope labeling of different metabolic pathways in mice, the authors identified a metabolic quality control mechanism that promotes mitochondrial health and liver regeneration. In healthy hepatocytes, fatty acids from adipose tissue reach the liver to fuel β-oxidation upon liver injury, thereby displacing cells with ETC-dysfunctional mitochondria. In these “malfunctioning” mitochondria, generation of acetyl-CoA through β-oxidation is inhibited, because the ETC is essential for this process. This leads to the accumulation of longer-chain fatty acids, blocking the breakdown of carbohydrates and eventually inhibiting the synthesis of acetyl-Coa from pyruvate Importantly, acetyl-CoA is not only used for energy production but also for epigenetic chromatin acetylation and as a precursor for cell membrane lipids, like phosphatidylcholine and phosphatidylethanolamine. The importance of cellular phospholipid synthesis in proliferating hepatocytes for liver regeneration has been recently demonstrated and was found to be influenced by the gut microbiota, which degrades and ferments complex carbohydrates, i.e. dietary fiber, to acetate.^3–5^

Mechanistically, the accumulation of excess lipids caused by dysfunctional ETC induces mitochondrial kinase PDK4 (*pyruvate dehydrogenase kinase 4*), negatively regulating the pyruvate dehydrogenase complex that generates acetyl-CoA from pyruvate. In parallel, excess lipids suppress the cytosolic enzyme ACSS2 (*Acyl-coenzyme A synthetase short-chain family member 2*), which converts acetate to acetyl-CoA.^1^ Wang and colleagues report that PDK4 inhibition actually increases the mitochondrial flexibility, allowing liver cells with dysfunctional ETC to use carbohydrates as an energy source, and, ultimately, to proliferate in the context of liver regeneration, with detrimental consequences.

The inability of “unfit” hepatocytes with a perturbed ETC to metabolize acetyl-CoA from pyruvate or acetate results in the privileged selection of hepatocytes with healthy mitochondria for proliferation, as acetyl-CoA production is prevented.

The authors further provide compelling evidence for the importance of transdifferentiation of cholangiocytes to hepatocytes during liver regeneration and convincingly show that a functional ETC is crucial during this process as well. By applying state-of-the-art methods like single nucleus RNAseq, the authors were able to further characterize TLPCs (transitional liver progenitor cells), the intermediate progenitor cell populations, and found an increasing expression of genes related to lipid metabolism along the transdifferentiation process. Moreover, cholangiocytes may have a profound impact on liver composition and zonation even under homeostatic conditions. Even though these findings are thoroughly controlled, the study is strongly based on preclinical animal models. This creates an inherent methodological bias, due to obvious species-specific differences between mice and men.

However, several questions remain to be answered, such as the role of this metabolic “quality control” mechanism in other cell and tissue types. Obviously, mitochondrial inflexibility is a double-edged sword, since it clearly is associated with obesity and other unfavorable conditions. Therefore, it would appear necessary to induce metabolic inflexibility exclusively in liver cells, if one aims at a direct therapeutical application. Of note, it is currently not clear how hepatic PDK4, the main beneficial factor for liver regeneration identified in this study, could be safely activated or how its expression could be increased in a tissue-specific manner. This could make a direct translational application based on PDK4 challenging, especially in patients with chronic liver damage. From a clinical perspective, it is noteworthy that insulin is well known to directly inhibit the transcription of PDK4. Therefore, measures to restore insulin responsiveness by exercise and dietary interventions could have beneficial side effects for mitochondrial health in the context of liver regeneration. Taken together, clinical data would be essential to determine the relevance of these processes for human liver disease, and it is currently unclear how new therapeutic interventions could be derived from the study.
